# Biotechnology in Food Packaging Using Bacterial Cellulose

**DOI:** 10.3390/foods13203327

**Published:** 2024-10-20

**Authors:** Maryana Rogéria dos Santos, Italo José Batista Durval, Alexandre D’Lamare Maia de Medeiros, Cláudio José Galdino da Silva Júnior, Attilio Converti, Andréa Fernanda de Santana Costa, Leonie Asfora Sarubbo

**Affiliations:** 1 Rede Nordeste de Biotecnologia (RENORBIO), Universidade Federal Rural Pernambuco (UFRPE), Rua Dom Manuel de Medeiros, s/n-Dois Irmãos, Recife 52171-900, Brazil; santosrmaryana@gmail.com; 2 Instituto Avançado de Tecnologia e Inovação (IATI), Rua Potyra, n. 31, Prado, Recife 50751-310, Brazil; italo.durval@iati.org.br (I.J.B.D.); alexandre.medeiros@iati.org.br (A.D.M.d.M.); claudio.junior@iati.org.br (C.J.G.d.S.J.); converti@unige.it (A.C.); andrea.costa@iati.org.br (A.F.d.S.C.); 3 Department of Civil, Chemical and Environmental Engineering, Pole of Chemical Engineering, University of Genoa (UNIGE), Via Opera Pia, 15, 16145 Genoa, Italy; 4 Centro de Comunicação e Desing, Centro Acadêmico da Região Agreste, Universidade Federal de Pernambuco (UFPE), BR 104, Km 59, s/n—Nova Caruaru, Caruaru 50670-900, Brazil; 5 Escola de Tecnologia e Comunicação, Universidade Católica de Pernambuco (UNICAP), Rua do Príncipe, n. 526, Boa Vista, Recife 50050-900, Brazil

**Keywords:** biodegradable packaging, biomaterials, polymers, industrial waste

## Abstract

Food packaging, which is typically made of paper/cardboard, glass, metal, and plastic, is essential for protecting and preserving food. However, the impact of conventional food packaging and especially the predominant use of plastics, due to their versatility and low cost, bring serious environmental and health problems such as pollution by micro and nanoplastics. In response to these challenges, biotechnology emerges as a new way for improving packaging by providing biopolymers as sustainable alternatives. In this context, bacterial cellulose (BC), a biodegradable and biocompatible material produced by bacteria, stands out for its mechanical resistance, food preservation capacity, and rapid degradation and is a promising solution for replacing plastics. However, despite its advantages, large-scale application still encounters technical and economic challenges. These include high costs compared to when conventional materials are used, difficulties in standardizing membrane production through microbial methods, and challenges in optimizing cultivation and production processes, so further studies are necessary to ensure food safety and industrial viability. Thus, this review provides an overview of the impacts of conventional packaging. It discusses the development of biodegradable packaging, highlighting BC as a promising biopolymer. Additionally, it explores biotechnological techniques for the development of innovative packaging through structural modifications of BC, as well as ways to optimize its production process. The study also emphasizes the importance of these solutions in promoting a circular economy within the food industry and reducing its environmental impact.

## 1. Introduction

Food packaging is a material or system used for the protection, containment, transportation, storage, and/or final use of semi-finished or finished food products. According to Jorge [1], these materials play a fundamental role in guaranteeing that products reach the end consumer in good quality, ensuring protection against physical, chemical, and biological factors, such as light, humidity, and the presence of microorganisms, in addition to controlling the internal atmosphere, thus slowing down processes such as oxidation and giving the product a longer shelf life. Yam et al. [2] also highlight that packaging conveys information and facilitates product use, saves time, and can contain products of different sizes. These concepts reflect multiple functionalities, ranging from preserving the integrity of products to facilitating their logistics and providing essential information for consumers.

**Figure 1:**
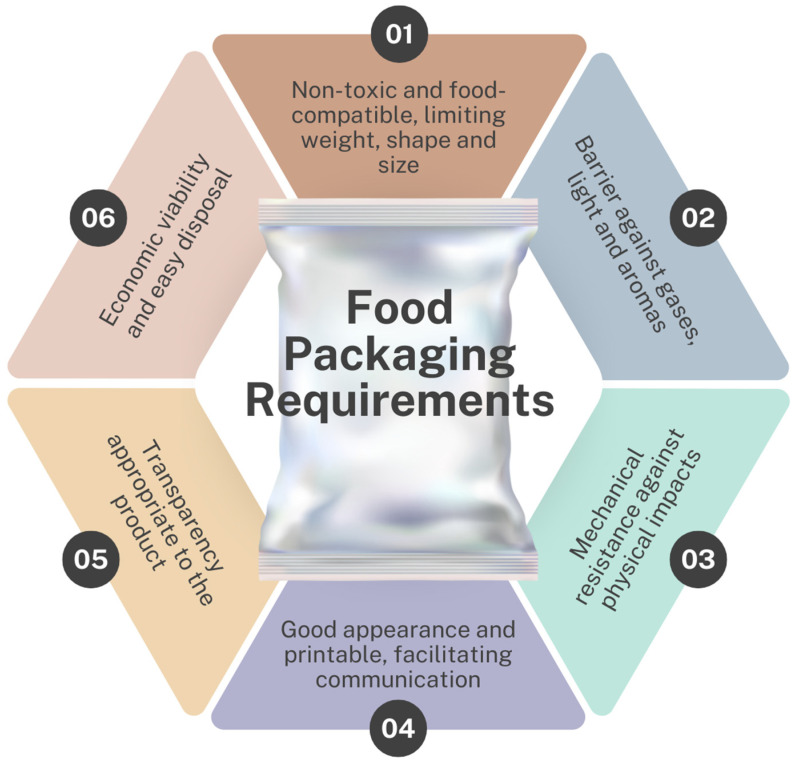
Main requirements that food packaging must meet.

Foods are vulnerable to deterioration caused by oxidation, microbial activity, and metabolic processes, which can be accelerated by factors such as temperature, moisture, and exposure to light [3]. Thus, packaging acts as a protective barrier, minimizing the effects of these factors and preventing contamination and physical damage. In addition, it guarantees the preservation of sensory and nutritional characteristics, ensuring food quality and safety. Therefore, packaging that is in direct contact with food must meet specific requirements, as illustrated in Figure 1 [4].

Materials such as glass, paper, metal, and plastic are widely used in the food industry. All of these materials have relevant characteristics; however, plastic is the predominant material among the packagings produced. The low cost, availability, and versatility confirm and drive the increase in demand for plastics in recent years. Excessive consumption of plastic is generating a serious environmental problem, especially due to its difficult degradation in nature [5]. In addition to being produced from raw materials derived from fossil sources, its production requires the use of a lot of energy. It is estimated that around 10% of the plastics thrown away reach the oceans through rivers, winds, and rainwater, fragmenting into micro and nanoplastics, which can be ingested by aquatic organisms, thus compromising the food chain [6].

Ponnusamy and Mani [5] stated that approximately 40% of total plastic production is directed toward the production of packaging, a value that tends to follow demand and population growth. Projections indicate that the global food packaging market could generate around USD 464 billion by 2028, a worrying figure considering the associated environmental challenges and impacts [7]. Given this scenario, it is crucial to promote the reduction in the use of petrochemical polymers, encouraging the transition to biodegradable options as well as the adoption of a circular economy model, which minimizes negative effects on the environment.

Biodegradable packaging in the food sector can be used in active, edible, or even modified atmosphere packaging systems [8]. According to the market analysis made by Mordor Intelligence [9], this segment is expected to generate around USD 105.26 billion in 2024, with projections to reach USD 140.66 billion by 2029. Packaging is often composed of biopolymers such as polylactic acid, polyhydroxyalkanoates (PHA), and polyhydroxybutyrate (PHB), whose global demand is expected to reach approximately USD 10,447.2 million [5]. Polymers with renewable and biodegradable characteristics are being used in primary, secondary, and tertiary packaging projects, aiming to reduce environmental impact. During the development of these solutions, it is essential to carry out tests to confirm the effectiveness in basic functions, such as preserving the product, preventing accelerated decomposition, and evaluating the interaction of the biopolymer with the food [4,5].

Technological advances and the growing search for sustainable solutions are driving the development of innovative packaging. The latest trends are aimed at creating materials that not only protect food but also actively interact with it and the environment [4]. In this context, biotechnology plays a central role, enabling the development of packaging that, in addition to being biodegradable and fulfilling its traditional functions, can release specific substances capable of extending the shelf life of food and increasing its level of preservation and freshness [8].

The choice of biodegradable, biocompatible, and non-toxic natural polymers is extremely important in the preparation of biotechnological packaging [10]. A promising biopolymer that combines these characteristics is bacterial cellulose (BC), an exopolysaccharide produced by certain strains of acetic acid bacteria, which exhibits important properties such as mechanical strength, water retention capacity, crystallinity, easy structural moldability [11,12,13], and degradability potential of 50% in 3 days and even 100% in more than 7 days [14]. Recent research reports positive responses of BC as a packaging film in the preservation of foods such as bread [15], fruits [16,17], dairy products [18], and fish [19]. These benefits include protection against pathogenic microorganisms and spoilage, reduction of oxidation and moisture loss, preservation of sensory properties, and the promotion of a sustainable packaging solution.

In view of this context, sustainability drives biotechnology-based innovation in the development of new biodegradable materials, which can play a crucial role in reducing the environmental impact of conventional packaging. The objective of this review is to provide an overview of conventional packaging and its impacts, the development of biodegradable packaging, the highlighting of BC as a promising biopolymer, and biotechnological techniques for the development of innovative packaging.

## 2. Conventional Food Packaging

Food packaging can be categorized into groups according to the type of material, degree of firmness, functionality, and purpose/use. This classification helps in choosing the best packaging to protect a given food. According to the degree of firmness, for example, packaging can be classified as rigid, semi-rigid, or flexible [20]. Rigid packaging maintains its original shape even after being filled with the product. Semi-rigid packaging maintains its shape only under light load, while flexible packaging is more malleable, easily adapting itself to the shape of the product [20]. Saha et al. [21] state that although flexible material is cheaper, it provides minimal protection against compression or puncture.

Depending on the functionality, it is also possible to group packaging into four categories, namely primary, secondary, tertiary (or distribution), and unit load (Table 1). Primary packaging directly encloses and protects the product, while secondary packaging groups multiple primary units together for easier handling [22]. Tertiary packaging simplifies bulk shipping of secondary packages, and unit load packaging combines multiple tertiary packages into a single load for efficient shipping and distribution.

In addition to this functional classification, packaging can be divided, according to its purpose, into consumer packaging and industrial packaging [23]. Consumer packaging, which includes primary and secondary packaging, is delivered directly to the end consumer. On the other hand, industrial packaging, consisting of tertiary packaging and unit loads, is used for storage and distribution to retail outlets. Each type plays a crucial role in the protection, conservation, and presentation of products, ensuring efficiency in the logistic chain and safety for the consumer.

Regarding materials, some are used to compose food packaging. Among the best-known and most used on an industrial scale are paper/cardboard, glass, metal, and plastic. However, regardless of the material, according to Adibi et al. [24], only virgin grades should be used to make direct contact with food.

### 2.1. Materials for Conventional Food Packaging

#### 2.1.1. Paper

Paper is valued for its printability, recyclability, and biodegradability, which make it ideal for the manufacture of food packaging [24]. However, its porous cellulose network makes it a very hydrophilic material incapable of forming an efficient barrier to gases and water, which are fundamental characteristics for any food packaging [25]. Nevertheless, it has been used as primary and secondary packaging for some foods such as microwave popcorn, tea bags, butter, fast food items, and many powdered foods [26].

Papers with specific characteristics are produced for use in packaging a wide variety of foods. Kraft paper and bleached Kraft paper are widely used for packaging flour, sugar, fruits, and some vegetables, while tissue paper serves as a wrapper for bread, sandwiches, and boxes. Tissues are commonly used for tea and coffee bags. Greaseproof paper and glassine have hydrophobic characteristics, the latter being ideal for coating baked goods or fatty foods. Finally, paperboard/cardboard is widely used during the transportation and storage of products such as beverages and fruits due to its strength and protective capacity [26,27,28].

Among the materials used for packaging, paper is considered the most sustainable in terms of disposal, as it is produced from plant fibers. However, paper production also presents challenges. More than 95% of paper is made from wood, while only about 5% comes from alternative sources, such as agricultural by-products [23]. Furthermore, the bleaching and purification processes of paper derived from vegetable cellulose generate chemical residues that are harmful to the environment [27]. This indicates that, although paper is easier to recycle and biodegradable, its manufacture still depends on forest resources, which can lead to environmental issues related to deforestation and intensive use of water and energy in the production process.

#### 2.1.2. Metals

Metal packaging, which conveys the idea of robustness and durability, is often used as primary packaging in the sectors of canned goods, beverages, and dairy products, among others [29]. Carbonated beverages such as beer, soft drinks, and wines, along with fruit and vegetable juices, seafood, fish like tuna, chocolates, and cookies, are some of the products commonly packaged in cans. The metal offers an excellent barrier against light, gases, and moisture, is recyclable and easily moldable into different shapes, withstands high temperatures, has a rigid structure for long-distance transportation, and allows for a variety of decoration possibilities [30]. Metal packaging is generally made of steel or aluminum, with steel being coated with chrome or tin [31]. Among the types of coated steel most commonly used for food packaging are tinplate (steel coated with tin on both sides), chrome-coated steel, and polymer-coated steel [26]. The coating on steel is due to its tendency to oxidize when exposed to moisture and oxygen [23].

Even after the steel has undergone a coating process, an additional layer must be applied to prevent elements of the metal surface from reacting with the food [30]. In addition to these precautions, it is important to consider the specific needs of each type of food. For example, food and beverage cans are often made of steel and aluminum, respectively, due to the distinct properties of each material and their respective suitability for the type of product to be packaged [30]. Failure to take care of these metal-food interactions can result in organoleptic changes, loss of vacuum, hydrogen swelling, metal concentrations above legal limits, or even perforation of the container, compromising the integrity of the product and consumer safety [32].

In terms of strength, aluminum has certain limitations when compared to steel, being less robust, but it is easier to mold seamlessly [33]. For this reason, aluminum is widely used in applications where the structural strength of the packaging is not critical, such as in the manufacture of beer and carbonated soft drink cans. In contrast, steel offers greater strength but does not respond well to the seamless deformation process, as it is not soft enough [33]. Thus, both materials have specific benefits and characteristics that must be considered according to the desired application. However, it is important to take into account some challenges associated with the manufacture of metal packaging, such as CO_2_ emissions during the production process, the possible migration of toxic chemicals from containers to food, and the risk of depletion of natural resources [30]. These factors highlight the need to balance the functional advantages of materials with environmental and food safety considerations.

#### 2.1.3. Glass

Glass has characteristics that make it attractive to both industries and consumers when used as packaging. According to Grayhurst and Girling [34], consumers often associate products packaged in glass with a perception of high quality. Among the advantages of glass are transparency, which allows the product to be viewed, impermeability, and resistance to all types of food without the risk of chemical substances migrating to the content [35]. According to Agarwal et al. [36], compared to plastic, glass has less effect on the flavor of food and protects it from external factors. In addition, this material is aseptic and thermally stable, withstanding processes such as hot filling and sterilization, and can be molded to facilitate brand recognition [1]. These properties make glass a reliable choice for the preservation and exhibition of food and beverage products.

Glass is manufactured from sand, soda, ash, and limestone [37]. Glass shards are also an important ingredient in production [23]. To mold and obtain this material in the desired shape, it is necessary to use temperatures above 1500 °C until complete melting [29]. This molding generates different types of packaging, with cylindrical shapes and narrow edges (bottles) or wide edges (jars) [37]. In addition, with regard to color, these packages can be colorless or pigmented through the addition of inorganic elements that are incorporated at the time of preparation [1]. Thus, the properties of glass and its versatility in terms of shape and color make it a versatile choice for food and beverage packaging, and it is commonly used for solid or liquid products, heat-treated products, or products packaged under pressure [37]. Products such as alcoholic beverages, soft drinks, juices, and condiments, including oils and tomato sauces, are often packaged in glass.

The reuse of glass containers, allowing for their repeated use in different contexts, highlights this material as an attractive option for packaging [1]. However, for glass to be considered a sustainable solution, it is crucial to evaluate the entire life cycle involved in its production. The glass manufacturing process requires significant energy resources, such as fossil fuels and electricity, in addition to generating greater air pollution compared to plastic [35]. As a result, glass production contributes to the emission of greenhouse gases, with an environmental impact that can vary depending on the energy matrix of each country [38,39]. Therefore, the sustainability of glass depends not only on its reusability but also on practices that involve cleaner and more efficient production.

#### 2.1.4. Conventional Plastic

Plastic is characterized by being a polymer that can be molded into a wide range of shapes through the controlled application of heat and pressure. In addition, it has important characteristics for the food industry, such as lightness, ease of handling and transportation, long durability, perfect sealing, resistance to weather and water, and low cost per use [40]. These functionalities are entirely related to the chemical structure of the plastic raw material and properties such as linearity, molecular weight and distribution, density, crystallinity, humidity, and temperature [41]. With this in mind, the arrangement and conformation of the constituting monomers are important factors in the individual characteristics of plastics, and these polymers can be classified, according to their linearity, as linear, branched, cross-linked, and networked [41]. Modulating certain properties of plastic polymers can directly impact their functionalities, especially in the design and development of packaging. For example, crystallinity, which refers to the degree of organization of polymer chains, has a significant effect on the characteristics of the film, such as its thermal and mechanical resistance [42].

Fossil raw materials, mainly oil, gas, and sometimes coal, are used almost exclusively in the production of synthetic plastics [40]. Polymerization and structural changes produce polymers such as polyethylene terephthalate (PET), polyvinyl chloride (PVC), polystyrene (PS), polypropylene (PP), and polyethylene (PE) [43]. For example, PET is a thermoplastic widely used in beverage bottles due to its transparency, lightness, strength, and ability to act as a barrier against gases [44]. PVC, in turn, is versatile and can be used in rigid or more flexible applications. In food, PVC often acts as a flexible film for preserving food in supermarkets or domestic kitchens [45]. PS is a low-density, cost-effective material known for its durability, low moisture absorption, and excellent thermal insulation properties [46]. Its transparency and strength make it a common choice for applications such as meat and fruit trays, fast food containers, and disposable cups [47]. PP is popular for its chemical resistance, ease of processing, high tensile strength, and ability to withstand high temperatures while also being aseptic, physiologically inert, and moisture-resistant, making it ideal for packaging applications such as containers and bottles [47,48]. Finally, PE is the most extensively used plastic in food packaging due to its favorable processability and excellent barrier properties. It possesses a diverse range of crystalline structures influenced by density and chain branching, and it is available in both high-density and low-density forms. PE is commonly utilized in plastic bags, packaging films, and containers, and it offers the advantage of heat sealability [47,49].

Although plastic has wide applicability and offers many benefits in the packaging sector, it generates significant environmental impacts throughout its life cycle, from the extraction of fossil raw materials to improper disposal. The accumulation of plastic waste in the environment, such as microplastics, affects ecosystems and human health. According to Cverenkárová et al. [50], 80% of plastic waste in oceans comes from terrestrial waste. To mitigate these effects, it is essential to understand the properties of plastics, their limitations, and their impacts while seeking more sustainable alternatives, such as biodegradable plastics or plastics from renewable sources, in addition to adopting reuse and recycling systems.

### 2.2. Impacts and Challenges Related to the Use of Conventional Plastic Packaging

Aggravated by population growth and consumerism, the use of conventional plastic packaging results in an alarming situation due to the generation of waste, whose accumulation impacts both terrestrial and aquatic environments. This impact is generally caused by incineration or slow degradation, which releases greenhouse gases, and by accumulation in the oceans, which unbalances marine ecosystems [51,52]. The degradation of non-biodegradable plastic of fossil origin, on a micro and nanometric scale, such as abrasion and photo-oxidation, is caused mainly by atmospheric agents in combination with microbial processes [6]. In marine environments, the improper disposal of these materials, combined with ocean currents, results in the formation of “floating islands” of plastic that release fragments along their paths, affecting marine fauna, an important source of human food [44]. In terrestrial ecosystems, microplastics can also infiltrate food chains, carrying associated chemicals and accumulating at various trophic levels, and they can have potentially harmful effects on human health and on other organisms [53]. Studies have identified microplastics in seafood, sea salt, and drinking water, as well as in the gastrointestinal tract of marine animals and the human intestine [6].

The packaging sector is a major driver of environmental pollution, as it is the largest consumer of conventional plastic on the market [27]. According to Jafarzadeh and Jafari [54], more than 300 million tons of plastic waste are generated annually in the world, part of which comes from disposable materials [55]. With this in mind, it is observed that the plastic packaging chain is still linear, with much of its value being lost in just one use [56]. Within this context, this issue may be related to poor waste management practices by major companies, both in the packaging sector and industries that heavily rely on packaging materials, such as the food industry. These companies often fail to implement effective recycling, reuse, and innovation strategies in sustainability, such as incorporating biodegradable materials into their processes. Furthermore, although some companies have systems for returning this packaging, only 2% of plastic waste is recycled to become new packaging, as reported by Defruyt [57]. Therefore, it is clear that, although there is some concern, the change is still very small compared to the size of the problem related to plastic waste, characterizing it as a constant challenge.

**Figure 2:**
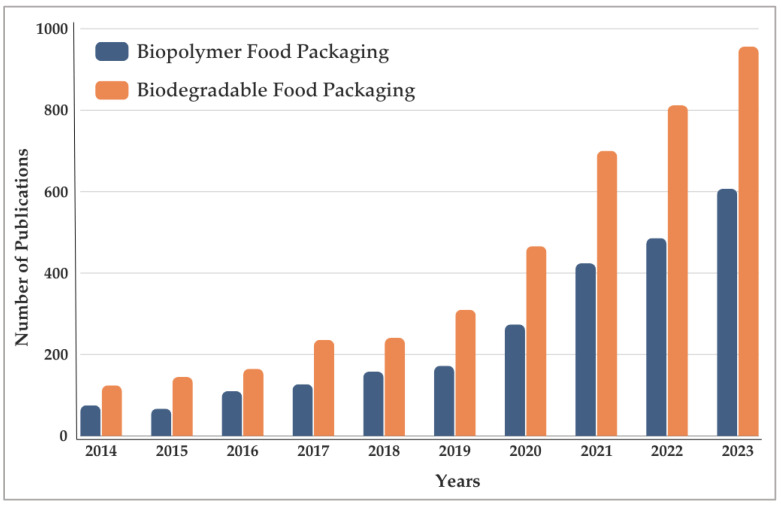
Number of publications in the last 10 years related to sustainable food packaging.

Hoque and Janaswamy [7] report that the global food packaging market is expected to reach approximately USD 464 billion by 2028. Considering that the food and beverage sector is one of the largest users and producers of packaging, this should be the starting point for reducing the use of petrochemical polymers in the environment and for the transition to biodegradable polymers. This sector has primary needs, such as long shelf life, food safety, and maintenance of the quality and sensory characteristics of products, and conventional plastic meets these demands [52,56,58,59]. However, in addition to environmental impacts, according to a report by the World Wildlife Fund (WWF), the Ellen MacArthur Foundation (EMF), and the Boston Consulting Group (BCG) [60], there is growing pressure and appeal from the public for industries to adopt a circular economy and use sustainable packaging [27]. Given this scenario, the search for alternatives for reusing or replacing petrochemical polymers has been increasingly studied, as evidenced by the increase in the number of annual publications in Google Scholar relating to the descriptors “biopolymer” and “food packaging” and “biodegradable” and “food packaging” (Figure 2).

## 3. Biopolymer Food Packaging

The evolution of packaging technologies has allowed the creation of more sustainable and innovative solutions, among which biotechnological packaging produced using biopolymers stands out. For the purposes of this research, biotech packaging is defined as a type of packaging developed using biotechnology principles to obtain biopolymers, i.e., using biological sources, such as plants or microorganisms [61]. In addition to being made of biodegradable material, the most interesting factor is that biotechnological packaging can easily incorporate innovative features: for instance, the ability to decompose more quickly, the effective preservation of products, and even the release of specific substances to extend the shelf life of food [61,62].

Biodegradable packaging is so called because it undergoes natural degradation through the action of microorganisms, including fungi and bacteria, which generates simpler and more natural components without causing harm to the environment. Biodegradation involves physical and chemical transformations in the material, which occur due to biological activity and can be influenced by some factors, including moisture, temperature, surface area, and hydrophobicity/hydrophilicity [62,63]. Therefore, when formulating an environmentally sustainable material, it is essential to consider these properties to ensure that biodegradation occurs effectively. In addition, seeking to reduce costs and improve mechanical properties is of great importance in the production of sustainable packaging, considering that conventional plastic packaging offers advantages in these requirements. To gain market share within the industry, it is insufficient for a raw material to be simply sustainable. It is essential to explore alternatives that not only remain cost-effective but also provide robust performance.

**Figure 3:**
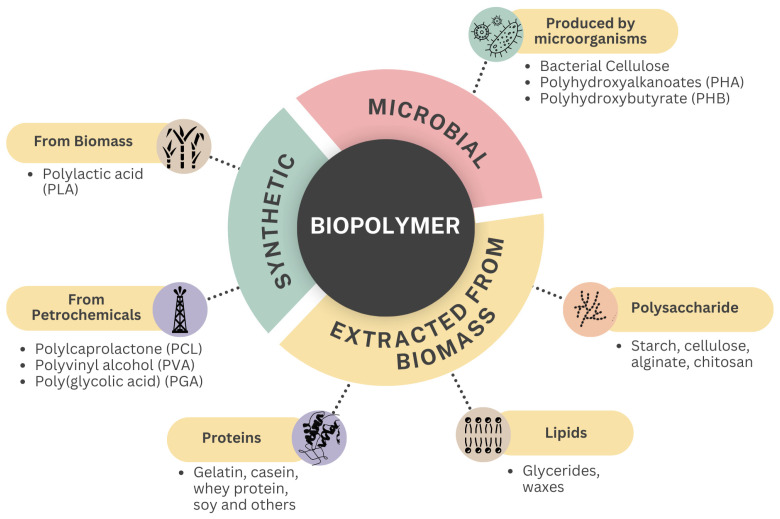
Biodegradable polymers classified by source. Image adapted from Baghi et al. [55].

Therefore, there has been a growing interest in biomaterials that meet these criteria, although challenges related to production costs and mechanical, thermal, and optical properties persist. Recent studies have focused on developing polymeric materials for packaging that successfully integrate sustainability, functional effectiveness, and innovation [64]. Polymers are categorized based on whether they are fossil-based or bio-based and whether they are non-biodegradable or biodegradable [63]. Based on this categorization, biopolymers can be divided into synthetic, natural (from biomass), or obtained through microbial fermentation processes [65] (Figure 3).

Biodegradable fossil polymers such as polycaprolactone (PCL) have a chemical structure formed by polyester chains, which makes them easily degradable by microbial action [66]. Although some of these polymers have good mechanical characteristics, including elasticity, tensile strength, and resistance to impact, their non-renewable origin represents a significant disadvantage due to the finiteness of fossil resources and the high application costs [67]. In view of this, many studies have focused on the development of polymers obtained from biomass, with the aim of developing more sustainable and environmentally friendly biotechnological packaging [59].

Synthetic biopolymers are obtained through modifications in the chemical structure of natural polymers, which give them characteristics such as biodegradability, biocompatibility, and mechanical strength [61]. Polylactic acid (PLA), for example, has emerged as a promising material for packaging fish and fruit [68,69]. Its production can occur through ring-opening polymerization of a cyclic dimer of lactic acid (lactide) or through polycondensation of D- and/or L-lactic acid [61]. There are efforts to replace PET with PLA in the production of bottles and films due to the similar performance of these polymers [70]. However, PLA has limitations due to its high cost, brittleness, and lack of gas barrier properties.

Bio-based polymers can also be obtained from biomass or produced by microorganisms [71]. Polymers derived from proteins, polysaccharides, or lipids offer an excellent foundation for packaging development due to their biodegradability, renewability, and natural abundance, making them a sustainable alternative to conventional materials [59]. Starch is an example of a polysaccharide that has been studied to produce packaging, whose plastic properties can be improved through modifications, the addition of reinforcing groups, or mixing with other polymers [72]. In addition, other polysaccharides, such as alginate and chitosan, have attracted interest. Chen et al. [73] observed that the incorporation of phenolic compounds into a sodium alginate film applied to freshly cut apples resulted in antimicrobial effects, reduced weight loss, and nutrient retention. Additionally, the alginate film exhibited good mechanical strength and effective UV-vis light-blocking properties. Miwia et al. [74] developed a chitosan-based film extracted from the biomass of *Tenebrio molitor* larvae, which successfully preserved the quality and texture of packaged bananas for up to 10 days.

The proteins most commonly studied for packaging applications are soy protein and gelatin [75]. Gelatin films have been extensively researched for packaging development, with the main challenge being to improve their vapor barrier properties and reduce their sensitivity to moisture [59]. Similar to gelatin, soy protein also faces these limitations when considered for packaging applications [75]. To overcome this, the incorporation of plasticizers or other additives is a feasible approach to achieve the desired properties. Wu et al. [76] discovered that adding zinc oxide nanoparticles to a soy protein film improved its tensile strength and elongation at the break while also reducing oxygen permeability. These modifications make it possible to effectively use environmentally friendly materials, enhancing their performance and meeting industry demands.

One promising alternative for creating biodegradable packaging is using microorganisms to develop polymeric films. A widely studied biopolymer in this area is polyhydroxyalkanoate (PHA), which is commonly used for making bottles, boxes, and films due to its beneficial physical and mechanical properties [77]. However, its large-scale production is limited due to high costs. To address this issue, researchers have been exploring the use of inexpensive substrates such as food waste, lignocellulosic biomass, and used cooking oil [78]. Notably, vegetable oil has been found to result in high PHA production [79].

Another promising example is bacterial cellulose (BC), obtained through a simple fermentative production process, which has been studied as packaging or part of composites in an attempt to reduce dependence on fossil resources [80]. Bacterial cellulose (BC) is well-known in Asian countries and can be found in the fermentation of kombucha tea [81]. However, similar to polyhydroxyalkanoates (PHA), the high production cost of BC is a significant challenge, but there is ongoing research to develop more cost-efficient methods. BC is a versatile biomaterial with commercial potential due to its natural purity, biodegradability, biocompatibility, and non-cytotoxicity [82]. Additionally, it can be produced from agricultural and industrial waste, contributing to the circular economy and environmental sustainability. Because it is biodegradable, BC is an alternative in the food packaging sector; however, there is a need to improve important properties, including mechanical resistance, which is necessary for product protection during storage and transportation.

## 4. Bacterial Cellulose in the Development of Food Packaging

The extracellular synthesis of biopolymers, such as polysaccharides, is a fundamental part of the physiology of certain bacteria. Their synthesis is the result of secondary metabolism, as a defense mechanism, or a way to remain close to the surface and acquire oxygen [83]. BC is an exopolysaccharide produced by certain strains of acetic acid bacteria, which has superior properties to polymers obtained from natural sources [11,12]. These properties can be optimized through biotechnological tools, allowing BC to replace different synthetic materials in specific applications [84]. Due to these advantages, the BC market has grown significantly, and according to reports by ProfShare [85] and Business Research Insights [86], it is expected to move between USD 1396.94 and USD 1495.71 million by 2031.

The use of BC covers several industrial segments, including paper and cardboard, food products, cosmetics, and composite materials [80,85]. However, efforts have been made to reduce the cost and optimize its production process through the investigation of new low-cost culture media, the discovery of new bacterial strains, and the use of synthetic biology tools to increase production yields or confer new properties to BC [87]. With these advances, it is expected that BC will become even more competitive and accessible, expanding its use in several sectors.

### 4.1. Molecular Structure and Properties of BC

**Figure 4:**
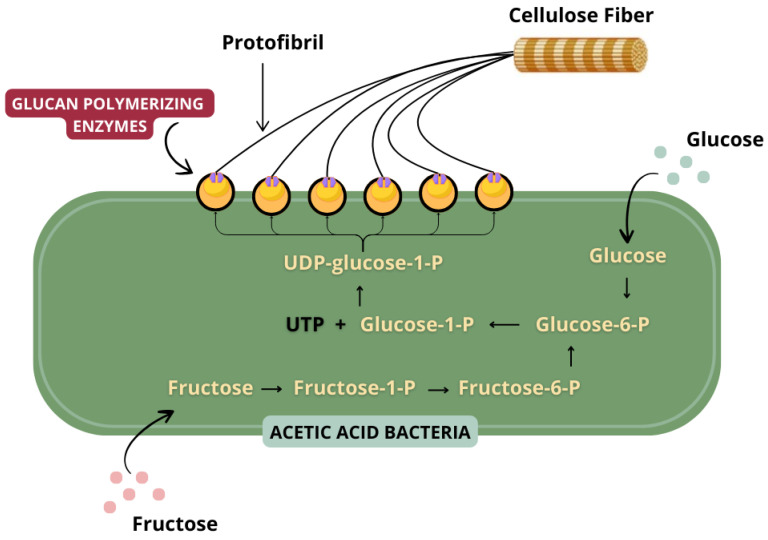
Schematic of the production of CB microfibers. Image adapted from Swingler et al. [92].

BC is a polysaccharide composed of repeating units of cellobiose, which is the product of the union of two D-glucose molecules through the β-1,4-glycosidic bond [88]. Compared to plant cellulose, it has a higher degree of purity, as it is not linked to other components such as lignin, pectin, or hemicellulose and exhibits a unique structural organization. The chemical structure is based on linear chains of β-1,4-glycosidic units that interact through intra and intermolecular hydrogen bonds, hydrophobic interactions, and van der Waals forces [89,90]. These β-glucose chains form regular protofibrils that aggregate to constitute nanofibrils assembled on a micrometric scale (Figure 4). The microfibrils, in turn, organize themselves into macrofibrils, which result in the formation of the cellulose fiber [91,92].

The mechanical strength, water retention capacity, easy structural moldability, and crystallinity of BC result from its chemical structure [13]. Due to the large amount of hydrogen bonding, along with a high presence of hydroxyl groups and oxygen atoms, polymerization leads to the stacking of chains, forming nanofibrils through van der Waals forces [93]. This structural organization contributes to the hierarchical order of the fibrils, which in turn provides BC with its remarkable mechanical strength [94]. The unique arrangement of these fibrils imparts specific characteristics to this polysaccharide, making it distinct in terms of its strength and functionality. These fibrils can reorganize after the application of stress. Rheological analyses revealed that BC gel fibers exhibit reversible behavior after mechanical stress, in the breaking of bonds and the formation of new cross-linked interactions [95]. This structural recovery is a key feature for various applications.

The strength of BC fibers is directly related to the arrangement of the fibrils. Khattak et al. [96] showed that compacted fibrils exhibit greater tensile strength but reduced flexibility. On the other hand, fibers with pores and a looser arrangement have greater elasticity but lower strength. Overall, due to the strong interfibrillar bonds in the ultrafine fibril network, BC exhibits high stiffness [97].

Another crucial factor influencing mechanical strength is the hydration level of BC. Chen et al. [98] reported that hydrated BC displays elastic behavior determined by the dynamics of water within the hydrogel. Conversely, the dry film is more rigid and exhibits higher tensile strength. This variation in mechanical behavior is due to the presence of water molecules, which facilitate interfibrillar sliding by weakening hydrogen bonds [99]. Benítez et al. [100] studied the relationship between hydration and toughness in BC and found that when the BC film is dry, it becomes more rigid and resistant to tension but less tough (more prone to breaking). On the other hand, it loses some of this strength when hydrated but gains flexibility and a greater capacity to absorb forces without breaking. In this context, strategies such as structural modifications are needed to balance toughness and strength.

The regular arrangement of microfibrils also ensures crystallinity between 85% and 90%, which reflects the high degree of structural order of the material [91]. Crystallinity can be quantified using techniques such as X-ray diffraction (XRD), infrared (IR) spectroscopy, and nuclear magnetic resonance (NMR), which provide the crystallinity index, expressing the degree of crystallinity as a percentage [101]. The high crystallinity of BC is one of the key factors contributing to its excellent mechanical and thermal properties, as well as directly influencing its optical characteristics.

Optical properties such as transparency are crucial in packaging development and are often evaluated based on low opacity values [102]. Cazón et al. [102] found that BC films exhibit good transparency compared to other biodegradable films, such as chitosan-polyvinyl alcohol. Due to this transparency and light transmission, BC has potential in the production of contact lenses [103]. However, besides transparency, opacity can also be advantageous as a barrier to ultraviolet (UV) radiation, an important factor since UV radiation contributes to lipid oxidation in foods [104]. Jang et al. [105] reported that BC films have transmittance values of 5.61 for UVA and 2.10 for UVB, demonstrating good UV protection. Thus, depending on the production process and the modifications applied, BC films can achieve the desired optical characteristics.

BC also exhibits excellent thermal properties, which are crucial for packaging production. Thermogravimetric analyses are essential for assessing the stability of the film under high temperatures by monitoring mass loss. Factors such as sample geometry, mass, compatibility, and heating rate influence BC’s thermal stability [106]. Jang et al. [105] reported that wrinkled BC film shows thermal stability with less degradation at 325 °C. Volova et al. [107], on the other hand, analyzed different carbon sources as substrates and found that these significantly affected the thermal characteristics of BC, with BC synthesized in a galactose medium exhibiting greater stability.

**Figure 5:**
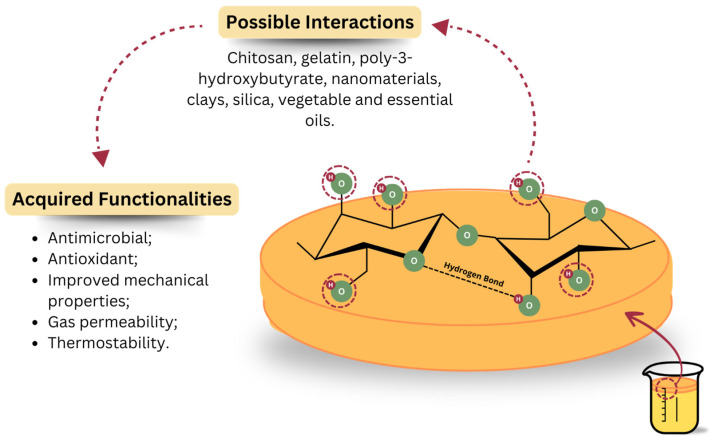
Possible interactions with OH groups and acquired functionalities. Image adapted from Grzybek et al. [109].

The arrangement of fibrils also provides a greater surface area, increasing the capacity for interaction with water molecules [90]. The abundance of hydroxyl groups (OH) and their strong bonding make BC highly hydrophilic, resulting in a high water retention capacity. This characteristic gives wet BC membranes the appearance of a highly swollen gel [91]. In addition, the reactive OH groups favor a variety of modifications, which can confer special functionalities to BC (Figure 5) [90,108].

Figure 5 illustrates the possibilities of combining BC with other compounds, as described by Grzybek et al. [109]. Chemical and structural modifications and different functionalizations have already been tested to improve the performance of BC [110] and produce more complex polymer blends or composites. A blend is a physical mixture of two or more polymers without chemical reactions between them, while a composite is composed of two phases, where at least one is a polymer (continuous phase), and the other is dispersed as filler or fiber (dispersed phase) [111,112].

Promising interactions between BC and antimicrobial compounds, such as essential oil extracts, nanoparticles, and organic acids, have already been observed, indicating their potential in the development of bioactive food packaging materials [14,113]. These bioactive compounds have demonstrated antimicrobial activity when incorporated into BC, which helps to extend the shelf life of food products. For example, Mesgari et al. [15] showed that cerium oxide nanoparticles incorporated into BC inhibited the growth of *Escherichia coli* and *Staphylococcus aureus* by more than 90%, with no observed cytotoxicity. Papadaki et al. [114] observed that incorporating oregano oil into an edible whey protein film, reinforced with bacterial cellulose nanowhiskers, imparted significant antimicrobial properties. This suggests that such combinations can create effective and safe antimicrobial packaging solutions. However, before making any changes to BC, it is crucial to consider the production conditions as well as the time and form of insertion of other substances to ensure the efficacy and stability of the resulting material.

### 4.2. Process Conditions for CB Biosynthesis

Microorganisms belonging to the genera *Rhizobium*, *Agrobacterium*, *Komagataeibacter,* and *Sarcina* are good BC producers [13,80]. *Komagataeibacter* spp. stands out due to their ability to convert ethanol into acetic acid and release large amounts of BC, being models for studies and industrial applications [87,108]. In particular, the species *Komagataeibacter xylinus* has stood out as an efficient substitute in the kombucha microbiota due to its high cellulose production capacity [115]. Furthermore, *Komagataeibacter hansenii*, cultivated in batch, demonstrated a high production yield, resulting in a biopolymer with advantageous properties, greater crystallinity, and good thermal properties [116]. The BC properties vary depending on the strain used, the carbon source, the culture medium, and the fermentation conditions [117].

When glucose is used as a carbon source, these bacteria follow a synthesis pathway composed of five main steps: (1) Phosphorylation of glucose into glucose-6-phosphate catalyzed by glucokinase; (2) Isomerization of glucose-6-phosphate into glucose-1-phosphate catalyzed by phosphoglucomutase; (3) Synthesis of UDP-glucose catalyzed by UDP-glucose pyrophosphorylase; (4) Release of UDP-glucose outside the cell as a monomer; (5) Polymerization of UDP-glucose through β-1,4-glycosidic bonds, which is catalyzed by cellulose synthase, with formation of CB (Figure 4) [87,89,91].

The glucose chain formed is expelled through small pores in the bacterial cell wall. These extrusions aggregate to form microfibrils, which, when accumulated, create a cellulose network, visible to the naked eye as a film on the surface of the culture medium [118], composed of 0.5 to 1% pure cellulose and 99% water [119]. It is noteworthy that cellulose can be expelled in two ways—as cellulose I, which occurs when extrusion is performed with stabilization and ordering of the microfibrils, or as cellulose II, which is formed in the absence of this stabilization—resulting in deterioration of the fibrils and, consequently, in a disordered structure [109].

Microbial fermentation can be performed statically or under agitation [108,120]. In static production, a simple method, the BC film forms on the surface of the culture medium after inoculation, floating due to the CO_2_ bubbles released by the cells. BC assumes the shape of the culture container and shows good characteristics of mechanical resistance, biocompatibility, and biodegradability [91]. However, this method has limitations, such as long cultivation time, low productivity, and uneven BC thickness due to the heterogeneous exposure of bacterial cells [121]. A quasi-static cultivation method can be used to mitigate these limitations, which includes continuous feeding of the culture medium and allows for increasing the process yield [116]. In production under agitation, the cells, in turn, are evenly distributed in the culture medium due to continuous mixing, ensuring greater yield and generating irregular structures instead of a film [122]. The high probability of the formation of negative mutants due to greater turbulence and shear stress is the challenge of this mode of operation, but there are modern reactors that allow overcoming such a drawback [89,123]. After obtaining CB, a purification step is necessary to remove impurities and remove bacterial cells [124].

As previously mentioned, some modifications can improve the characteristics or confer specific properties to BC membranes [13,14]. They can be made in situ, i.e., carried out during fermentation or with the synthesis of particles inside the membrane, or ex situ, i.e., the particles are added after fermentation [83]. Studies demonstrated that modification with nanoparticles and plant extracts can enhance the properties of BC, conferring therapeutic and antioxidant characteristics [125,126]. In particular, the use of vegetable oils or nanoparticles as antimicrobial agents is of great interest to the food industry [127,128,129]. Atta et al. [130] observed that the incorporation of silver nanoparticles into BC confers antimicrobial activity and increases the shelf life of foods, making BC a promising option for packaging.

In addition to the modifications required to develop films with specific properties, it is crucial that the operating conditions, including pH, temperature, oxygen level, and composition of the medium, are optimized to meet the needs of the microorganism and ensure high production yields. Cielecka et al. [11] observed that both pH and carbon sources significantly influence the strength of BC films. They found that membranes produced at a pH between 5 and 6 exhibited greater elongation, while those obtained at a pH above 6 showed reduced elasticity. In fact, each bacterial strain has an ideal pH and temperature range for its growth. In general, the optimal temperature range for growth is between 25 and 30 °C, while the optimal pH value varies between 4 and 7 [108,120]. The oxygen level is another crucial factor in microorganism growth, as they are aerobic. Lahiri et al. [93] reported that maintaining 10% dissolved oxygen saturation in fed-batch cultivation results in the highest BC yield.

The culture medium, in turn, must contain an easily assimilable energy source in sufficient quantity for BC production. Moreover, the intended application of the BC should be considered when selecting the carbon source. Cielecka et al. [11] studied the use of glucose compared to glycerol as a carbon source in BC production. They found that glucose resulted in greater film elasticity. The authors suggest that this variation in the tensile properties of BNC membranes produced with different carbon sources can be attributed to the crystallization process.

The study of alternative carbon sources for BC production has been more frequent. Industrial-scale production faces some problems related to the high cost of the culture medium and low yield. Therefore, finding economical culture media that provide the necessary nutrients to optimize this process is of great importance.

### 4.3. Cultivation Media and Use of Industrial Waste

The standard medium for BC production is the chemically defined Hestrin-Schramm (HS) medium, composed of glucose, yeast extract, and peptone as nitrogen and carbon sources [108]. However, these components are expensive, representing 50% to 65% of the total cost of BC production, which limits its large-scale application [110]. Therefore, the search for more economical alternatives has been a frequent topic in scientific research. It is known that BC-producing strains may require different sources of carbon and nitrogen. Some strains of *Komagataeibacter* produce more BC when using glycerol and sucrose as carbon sources; for nitrogen, sources from whey and rotten fruits result in a higher yield compared to the HS medium [121].

The use of industrial waste in the production of BC offers an alternative route that helps to reduce both pollution and costs associated with waste management [13]. Several substrates from industrial waste can generate BC at a similar or higher yield than the HS medium (Table 2). Within the food industry, the fruit pulp, dairy, and brewery sectors have shown good results in BC production [84,131,132]. However, it is important to pay attention to the purpose of the material since the composition of the medium determines its morphology and physical properties [108].

### 4.4. Application of BC Film as Food Packaging

Due to the diverse properties obtained using different substrates and modifications in the chemical structure, BC can be applied in several sectors, among which the cosmetic [142], environmental [143,144], pharmaceutical [145], and food [17] sectors stand out. In the food sector, BC is tested in several ways—as a thickening agent, gelling agent, fat replacer, and packaging material [91,146,147]. Recognized as safe by the US Food and Drug Administration in 1992, BC is accepted as a food ingredient or additive [91], which further boosts its application in this context.

The versatile applications of BC in food packaging have been extensively demonstrated in several studies. These include its use as an edible film, serving as a protective barrier while being ingestible [148]. BC is also used in the preservation of fresh foods by reducing moisture loss and extending shelf life [19], as antimicrobial packaging when combined with agents that inhibit pathogen growth [15,17], as a biodegradable film, offering a sustainable alternative to conventional plastics [14], and as breathable packaging, regulating gas exchange to better preserve perishable foods like fruits and vegetables [89].

For packaging development, it is crucial to analyze mechanical, optical, and thermal characteristics, in addition to barrier and permeability properties. Among the mechanical characteristics, tensile strength (TS), percentage elongation at break (%E), and Young’s modulus (YM) allows for predicting whether the film will withstand transportation, handling, and storage, common in the food industry [81]. With regard to barrier properties, water vapor permeability assesses the amount of water vapor that passes through the membrane, preventing undesirable food changes, while water retention capacity provides information on the material moisture absorption [149,150].

In food packaging, factors such as appearance, accessibility, ease of use, and disposal play a critical role in consumer communication and interaction. Optimizing the properties that influence these aspects is of paramount importance. Zahan et al. [14], when observing that a BC-based material was degraded by 50% in 3 days and 100% in 10 days, also demonstrated the importance of biodegradability time, reinforcing the need to replace conventional polymers with biodegradable materials. The degradation rate of biocellulosic material can be influenced by factors such as molecular weight, crystallinity, composition (including structural modifications), and pH [151]. To accelerate degradation and reduce decomposition time, treatments using hypochlorite, nitrogen dioxide, periodate oxidation, and 2,2,6,6-tetramethylpiperidine-1-oxyl radical (TEMPO) oxidation have proven effective without altering the polymer structure [101]. Jang et al. [105] also report that the incorporation of polymers, such as guar gum, enhances biodegradability. The discoveries support the potential for enhancing biocellulose formulation for more sustainable applications.

When it comes to food packaging, visual appeal plays a critical role in consumer communication. Transparency can significantly influence consumer perception, especially for products where visual presentation is key. However, it is important to consider the nature of the product, as UV light penetration may impact food quality by triggering chemical changes like lipid oxidation [152]. Consequently, a film that not only allows product visibility but also acts as a barrier against UV rays becomes highly advantageous compared to conventional packaging materials [81]. The modifications made to BC in order to achieve or optimize their properties were demonstrated in some studies (Table 3). Silva et al. [153] developed films using nanofibrillated BC and cashew gum, which showed improvements in tensile strength, solubility, decreased permeability, and elastic modulus as the amount of BC increased. Dhar et al. [154], in turn, developed a BC film with graphene oxide and sugarcane residue with improved mechanical and crystallinity properties.

Among the various modifications, it is worth mentioning the development of bioactive packaging, which helps to extend the shelf life of food by eliminating or emitting specific compounds [59]. This bioactive packaging can be classified into three main categories: absorbers (which eliminate unwanted compounds), release systems (which emit beneficial compounds), and other systems [159]. BC, as a polymer that easily absorbs antimicrobials and antioxidants, can release these compounds during storage, contributing to maintaining the quality of food. Jebel and Almasi [150] incorporated zinc oxide nanoparticles into BC, providing an antimicrobial effect. The release of these nanoparticles was optimized with the use of ultrasound, demonstrating efficiency against *Staphylococcus aureus*. In a study carried out by Malheiros et al. [160], the use of antimicrobial peptides, namely bacteriocins produced by *Lactobacillus sakei* subsp. *sakei* 2a, in CB film, ensured the inactivation of *Listeria monocytogenes* for 5 days [160]. Antimicrobial components, such as bovine lactoferrin, together with CB and sausage casings, allowed obtaining a film with bactericidal activity [161]. Other examples can also be seen in Table 3.

The effectiveness of films in food preservation is evident through their ability to extend shelf life while maintaining the food’s sensory and quality properties. For instance, Atta et al. [130] demonstrated that oranges and tomatoes packaged with BC/Ag nanocomposites retained acceptable sensory characteristics, such as odor and color, across different storage temperatures for up to nine weeks. In the case of lipid oxidation-prone products like cheese, greater attention is required. Vásquez et al. [162] showed that after 60 days of cheese storage, the use of a laminated BC and chitosan film enriched with grape extract resulted in a 67.3% reduction in lipid oxidation. These findings underscore the effectiveness of sustainable packaging materials in preserving critical aspects of food quality, ensuring both consumer satisfaction and food safety.

### 4.5. Manufacturing of Bacterial Cellulose Packaging

After defining the microorganism, culture medium, and ideal conditions, the BC production process can begin. Following inoculation, the production time can vary from 1 to 14 days, depending on controlled variables [93]. Purification is necessary afterward to remove living cells, typically achieved using solvents like NaOH at high temperatures. In the final stages, the process must be adjusted to ensure that the BC reaches the desired final form. Various techniques are then employed to convert BC into packaging material, such as layer-by-layer assembly (LBL) and casting, the latter being the most used in laboratory settings.

According to Fotie et al. [163], the LBL technique involves depositing a thin layer of a substance onto a surface and repeating this process until the film reaches the thickness necessary to provide the required mechanical properties and barriers against gas and moisture. This method allows for alternating layers with other materials, such as chitosan, enhancing its versatility for coating various structures like containers, trays, and bottles. Furthermore, multilayer packaging, with nanocellulose at the core and plastic film on the surface, has demonstrated significant commercial potential [164].

In the casting method, BC is dissolved in a suitable solvent and poured into a pre-determined mold or Teflon-coated Petri dish. After drying, which involves using air dryers like ovens or tray dryers, the solvent evaporates, leaving a polymer layer that adheres to the mold, a crucial step in obtaining the appropriate microstructure [129]. Although commonly used in laboratory settings, this method has also been tested for continuous production. Freitas et al. [148] developed an edible film using BC, palm oil, and tomato puree through bench-scale casting and continuous casting, resulting in a film with reduced water vapor permeability.

## 5. Future Perspectives

Although there are many studies focused on BC biosynthesis from industrial waste, emerging topics still require further investigation. The application of nanotechnology in the development of BC-based food packaging, for example, has been gaining ground in research. Nanoparticles can provide innovative properties to packaging, such as greater mechanical strength, thermal conductivity, and improved antibacterial characteristics [165]. One example is the use of zein nanoparticles incorporated into microbial nanocellulose, which resulted in nanocomposites with desirable characteristics for this purpose [166]. Bakouei et al. [167] developed a bacterial nanocellulose film incorporated with zinc oxide nanoparticles that showed a significant reduction in water vapor permeability, enhancing the film’s barrier properties. Additionally, its use as packaging material led to a noticeable reduction in microbial spoilage in bread, improving its shelf life and safety. Similarly, a composite film based on BC, gellan gum, and TiO_2_ and CuO nanoparticles demonstrated protection against softening and decay in freshly cut bell peppers during storage [168]. In addition, nanoparticles can form, together with BC, sensory useful for controlling the quality of products to be packaged, such as sensors for detecting lactate as a quality parameter in dairy products [169,170,171]. Despite this potential, the field is still largely unexplored, with challenges related to legislation, costs, consumer acceptance, cytotoxicity, and material migration, which need to be better understood. In particular, the migration of nanoelements and other hazardous food additives is a major concern [3]. This is an important issue in food safety.

Despite significant advances in optimizing CB production conditions and improving CB properties, studies addressing economic viability and scalability are still limited. Gomes et al. [116] observed an increase in CB production by a strain of *Komagataeibacter hansenii*, with improvements in thermal properties and high crystallinity after two successive batches. Increased productivity by microorganisms can be achieved, for example, through nutritional manipulation [172]. For industrial applications, the optimization of reactors, whether continuous, semi-continuous, or discontinuous, together with the use of waste as a source of nutrients, offer promising alternatives, which still need to be further explored. New low-cost methodologies that guarantee high yield need to be tested for large-scale applications. One example is the pulverization of biomass nanofibers during BC biosynthesis, which allowed for a yield 1.4 times that of pure BC and excellent mechanical and crystallinity properties [173]. In addition, ways to reduce the energy for the production and processing of these materials are important [3]. The scarcity of studies focused on scalability and production costs limits the understanding of the real commercial potential of BC, reinforcing the need for more research focused on these crucial aspects.

Within this industrial aspect and in the face of environmental concerns, the search for reducing waste of natural resources and for efficient reuse becomes a priority. According to Vinci et al. [38], the transition from a linear production system to a circular model could be a promising solution. This movement would make it possible to mitigate negative economic effects by creating businesses and generating jobs, in addition to encouraging the transformation of traditional systems. Thus, the implementation of processes that reuse and value waste, instead of discarding it, would generate not only environmental gains but would also reduce operating costs, promoting sustainability and new sources of revenue for companies.

Another relevant aspect is the choice of the product to be packaged since it influences the effectiveness and future development of packaging materials. Most studies on CB films as packaging focused on fresh products, mainly meat and fruit, as shown in Table 3. However, exploring new types of food, such as dairy products, can open doors to different industrial applications. Expanding this range of uses is essential to consolidate CB as a viable and competitive alternative, especially in sectors that require packaging with specific characteristics.

## 6. Conclusions

This review highlights the great potential of BC as a material for biotechnological packaging. Biodegradability and biocompatibility, especially its safety for food-contact uses, make BC a promising solution for sustainable packaging in the food industry. The exceptional mechanical strength, flexibility, and tensile properties of BC are mainly due to its unique nanofibrillar structure, which gives it an advantage over other biopolymers that require additives to achieve similar properties. Additionally, various recent studies demonstrate that BC can be easily modified to improve key packaging features. Unlike plant-derived cellulose, BC is highly pure and does not require extensive chemical treatments to remove impurities. However, its widespread application remains a challenge despite its relatively simple fermentation process, highlighting the need for more research into industrial feasibility. Lastly, nanotechnology stands out as a promising path for advances in the development of biotechnological packaging. It is essential to conduct further studies on food safety, functionality, and degradation capacity to ensure an effective and safe transition to these new materials.

## Figures and Tables

**Table 1:** Classification of packaging according to the functionality: category, concept, and examples.

Category	Concept	Examples
Primary	Packaging comes into direct contact with the product, with the function of containing and preserving it; it guarantees food safety and extends the shelf life of food.	Beverage can; juice box; plastic cheese packaging.
Secondary	Packaging encloses one or more primary packages and protects them from damage during transportation and storage; it also facilitates transportation.	Shrink wrap and a plastic ring connector that joins two or more cans together.
Tertiary	Packaging is larger and involves secondary packages; it protects the product during distribution and ensures efficient handling.	Cardboard box
Unit load	A group of tertiary packaging is gathered in a single unit; it facilitates mechanical handling, transportation, and storage of large quantities of products.	Pallet of cardboard boxes

**Table 2:** Different wastes used in media for BC production, fermentation conditions, fermenting microorganisms, and yield.

Waste	Fermentation Conditions	Microorganism	Yield (g/L)	Reference
Canning industrial waste (pineapple core)	Static conditions for 15 days at room temperature	*Komagataeibacter xylinus*	2.52	[131]
Kitchen waste	Static conditions for 10 days at 30 °C; inoculum: 10% (*v*/*v*)	*Komagataeibacter rhaeticus* K15	3.69	[133]
Mango peels	Static conditions for 11 days at 30 °C; inoculum: 6% (*v*/*v*)	*Achromobacter* S33	1.22	[134]
Agro-industrial waste of asparagus peel	Static conditions for 25 days at 30 °C; inoculum: 10.5% (*v*/*v*)	*Komagataeibacter rhaeticus* QK23	2.57	[135]
Molasses	Static conditions for 3 days at 30 °C; inoculum: 5 × 10^−6^ cells/mL	*Pseudomonas* sp. BC6	9.3	[136]
Dairy wastewater and whey	Static conditions for 15 days at room temperature	*Bacillus velezensis* FZB42	31.9	[137]
Confectionery waste	Static conditions for 11 days at 30 °C; inoculum: 10% (*v*/*v*)	*Komagataeibacter sucrofermentans* DSM 15973	5.7	[138]
Pear peel and bagasse	Static conditions for 7 days at 30 °C; inoculum: 9% (*v*/*v*)	*Komagataeibacter rhaeticus M12*	10.49	[139]
Potato tuber	Static conditions for 7 days at 28 °C; inoculum: 2 × 10^5^ CFU/mL	*Komagataeibacter xylinus* ATCC 53524	4.3	[140]
Pineapple peel	Static conditions for 14 days at 18–30 °C; inoculum: 10% (*v*/*v*)	*Komagataeibacter xylinus*	3.8	[141]

**Table 3:** Films made from bacterial cellulose (BC) for use as food packaging.

Modification in CB	Bioactive Action/Improved Properties	Packaged Product	Reference
Incorporation of cerium oxide nanoparticles	Antimicrobial action; inhibition of the growth of *Escherichia coli* and *Staphylococcus aureus* by 93.7% and 98.0%, respectively	Bread	[15]
Infusion in palm oil, cinnamaldehyde and thyme essential oil	Active release of cinnamaldehyde (antimicrobial) molecules in vapor form as a green preservative; maintenance of high intrinsic degradability; reduction of gas permeability; increase in elasticity and transparency	Strawberry	[16]
Film formation with chitosan and incorporation of ε-polylysine	Increased antibacterial performance; improved tensile strength and thermal stability	Tilapia	[19]
Esterification with cyclic anhydride	Antimicrobial effect on the film surface and reduction of water vapor permeability	Strawberry	[155]
CB hydrolyzed with sulfuric acid and incorporated with carnosic acid and ε-polylysine	Inhibition of spoilage microorganisms; delay of lipid oxidation; good mechanical properties	Fresh cheese and mozzarella	[18]
Ex-situ impregnation with olive and ginger oils	Antimicrobial action against *S. aureus*, *Pseudomonas aeruginosa*, *E. coli*, *Candida albicans,* and *Trichosporon* sp.; prevention of spoilage	Orange and tomato	[17]
Gelatin and CB nanocomposite film crosslinked with cinnamaldehyde	Antibacterial activity against *S. aureus* and *E. coli*	-	[156]
Silver-decorated bacterial cellulose nanocomposites	Antimicrobial action against *S. aureus*, *P. aeruginosa*, *E. coli*, *C. albicans* and *Trichosporon* sp.	Orange and tomato	[130]
Modification with 2,2,6,6-tetramethylpiperidine-1-oxyl containing thymol and anthocyanin-rich purple potato extract	pH sensitivity and colorimetric responsiveness to volatile ammonia; good antibacterial and antioxidant activities	Shrimp	[157]
Modification with clove essential oil	65% reduction in microbial growth; improved mechanical and thermal properties	-	[158]

## Data Availability

No new data were created or analyzed in this study. Data sharing is not applicable to this article.
